# Assembly-style making: How structured making serves as an on-ramp to creativity and engineering design

**DOI:** 10.3389/fpsyg.2023.1120186

**Published:** 2023-06-09

**Authors:** Sarah Lukowski, Megan Goeke, Bette Schmit, Marjorie Bequette

**Affiliations:** Science Museum of Minnesota, Saint Paul, MN, United States

**Keywords:** makerspace, family learning, informal learning, creativity, early engineering

## Abstract

Makerspaces, workspaces where families can explore materials and tools collaboratively, can provide an opportunity for creative expression and early engineering learning in community spaces. The present study examined a cardboard-focused museum makerspace that included an assembly-style activity. Assembly-style making uses instructions to support makers. Such activities have been critiqued as limiting creativity and engineering thinking. However, makers who are less comfortable in makerspaces may benefit from assembly-style activities helping to scaffold their entry into the space. We explored these criticisms and potential benefits of assembly-style making through developing case studies of video data taken by families in a makerspace. Visitors made creative and personally meaningful creations when engaged in assembly style making. Moreover, assembly-style making mediated a family less comfortable with making to get started in the space alongside ample evidence of families following engineering design processes. Contrary to popular belief, assembly-style making offers an important support to novice makers, without eliminating creativity and engineering design processes, and should be considered in the mix of activities available in makerspaces to support makers of all levels of comfort in making.

## 1. Introduction

Drop-in makerspaces increasingly act as spaces of informal, out of school, early engineering learning (Martin, 2015; Children’s Museum Pittsburgh & Institute of Museum and Library Services (IMLS), 2017). As more community spaces consider developing makerspace programming, a key question is the mix of activities available and how program design relates to potential learning outcomes (e.g., Marcus et al., 2021). In this brief research report, we share findings from case studies in a cardboard-focused museum makerspace. The present study centers the experiences of local Black, Hmong, and Indigenous families, groups that have been historically marginalized in the maker movement and literatures on family learning in makerspaces (see Vossoughi et al., 2016 for review). We examined interactions around ‘Gravity Racer’, which is an assembly-style activity that pairs instructions for building a cardboard vehicle with a ramp for testing. We developed case studies to consider:

1. Do families evidence creativity when engaging with an assembly-style activity (Gravity Racer)?
2. How might Gravity Racer support groups that feel less comfortable (more novice) with making?
3. How does engineering thinking emerge within the frame of interaction with Gravity Racer?

Recent research has provided insight into the learning outcomes possible in maker activities. Bevan et al. (2020) provided a framework for noticing and documenting learning in makerspaces. They identified five broad areas: initiative and intentionality, problem solving and critical thinking, conceptual understanding, creativity and self-expression, and social and emotional engagement. Each dimension had unique behaviors suggestive of learner progress, such as in the problem solving dimension where learners might iterate on their creation, seek ideas or tools to solve problems, and develop workarounds. Though developed with educators and students, the learning dimensions and behaviors associated with them also expand the possibilities of what behaviors constitute family learning in makerspaces.

Beyond varied learning dimensions, activity types may also lend themselves to different educative values to support the expertise of the maker. Here, Bevan’s (2017) taxonomy of maker activities - assembly-style, creative construction, and tinkering - provided an additional lens for considering differences across making activities. Assembly-style activities share what and how something should be made, typically through provision of step-by-step instructions. Assembly-style activities may support the development of material and tool fluency, an essential step for novices to a particular maker practice to grow in skill and confidence in making. Creative construction and tinkering may support progressively more creative and self-initiated problem solving within making. These more open-ended styles were hypothesized to support maker agency and more authentic learning experiences (Dougherty, 2013; Martin, 2015).

Given the hypothesized potential limits on creativity and problem solving, not all informal scholars or practitioners feel comfortable with assembly-style activities. Concerned scholars voice that more structured maker activities may limit engineering learning potential and learner agency. These concerned voices paint a picture of children producing identical “tchotchkes” as a result of following predetermined, step-by-step instructions (e.g., Blikstein and Worsley, 2016; Davies, 2017). To allow for authentic engineering design cycles to occur and for youth to make items that are personally relevant, some makerspace designers have followed varied advice including creating entirely open-ended makerspaces (Clapp et al., 2017) or hiding away example creations (MakerEd, 2015). The current study sought to explore these concerns by examining an assembly-style activity across three dimensions: creativity, the interactions of novice makers, and engineering thinking, each described below.

Creativity in makerspaces is marked by playful exploration, responding aesthetically to the materials, connecting to personal interests, and using materials in novel ways (Bevan et al., 2020). By closely examining the processes and products of families interacting in the makerspace, we interrogated the extent to which an assembly-style activity limits opportunities for creative expression, or conversely, evidenced creativity. Potential benefits and downfalls of creative constraints have been documented in a wide range of literatures beyond makerspaces (Medeiros et al., 2014; Roskes, 2014; Acar et al., 2019). Perhaps more closely aligned with the learning potential of makerspaces, long debates considering the merits of didactic versus discovery approaches to learning evidenced that structured activities can support learning (Mayer, 2004; Kirschner et al., 2006), though others continue to find advantages to exploratory over didactic approaches (Bonawitz et al., 2011). These literatures hint at the possibility that creativity and structure are not related in a strictly linear fashion where more structure always results in less creativity. What might this look like in assembly-style making practices?

In contrast to the perceived limits on creativity, a benefit of assembly-style activities may include supporting novice makers in learning new practices (Bevan, 2017). Improved material or tool fluency relies on novice makers getting started in the space. Instructions in an assembly-style activity may be an important scaffold to getting started. However, how family groups less comfortable with making get started together is not well characterized. New evidence suggests that emerging engineering interest may act as a family-level phenomena (Pattison et al., 2020), suggesting that social interactions between family members play a role. Whereas some practitioner guides, such as the Youth Makerspace Playbook, provided scripts that discourage using instructions to overcome uncertainty in a makerspace (MakerEd, 2015, p. 72), the present study investigated a different approach in a makerspace that included an assembly-style activity.

Finally, as a form of early informal engineering education, there is interest as to whether makerspace activities support exposure to and early practice of engineering design processes. The engineering design process for young learners (plan, create, test, and iterate) is meant to echo the practices that engineering professionals follow to solve problems (Moore et al., 2014; Major, 2018). Assembly-style activities have been criticized as potentially limiting engineering thinking by having a set of instructions that diminishes a visitor’s need to plan, iterate and problem solve on their own (American Society for Engineering Education, 2020). However, Gravity Racer was designed with the intention that the activity hinted at the possibility of following the engineering design process. Visitors are supported in their plan (icon-based instructions for making a car) before having the opportunity to create (families create a vehicle), test (families can test their vehicle on the ramp) and iterate (families improve on their vehicle design). The potential for visitors to follow such a design process within the designed elements of the activity does not mean that families follow such a process. Thus, we also sought to document how families approached Gravity Racer as an assembly-style activity suggestive of engineering design processes.

Combined, the present study explored new frames for considering the benefits and limitations of assembly-style maker activities. The current study primarily examined video data of family engagement in a makerspace to develop case studies to interrogate Gravity Racer, which was designed as an assembly-style activity, along the dimensions of (1) creativity, (2) the approach of novice makers, and (3) engineering thinking. Given how widespread questions around the value of assembly-style activities run among makerspace educators and designers (MakerEd, 2015; Blikstein and Worsley, 2016; Clapp et al., 2017; Davies, 2017) developing cases that evidence creativity and engineering thinking within the frame of assembly-style activities is an important contribution in expanding our understanding of family learning in makerspaces more broadly.

## 2. Materials and methods

### 2.1. Cardboard City exhibition and gravity racer

Cardboard City was an indoor makerspace exhibition at the Science Museum of Minnesota. It featured a mix of activity styles within a city theme. The space was relatively unfacilitated; that is, while there were substantial supports needed to maintain materials and cleanliness of the space, a facilitator was not leading the visitors through the activity.

**Figure 1:**
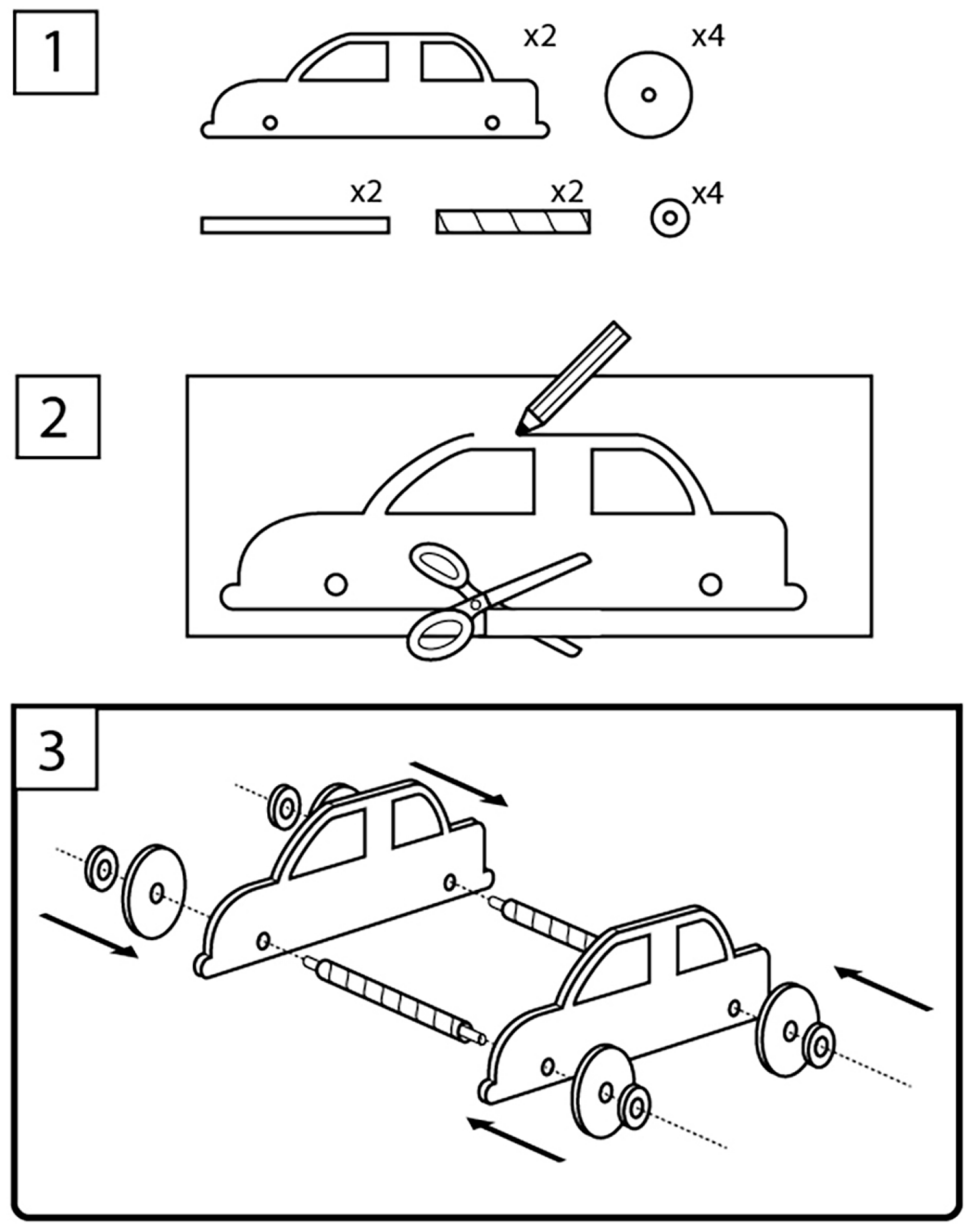
Icon-based Instructions for the Gravity Racer activity. The graphics depicted assembly-style instructions for making a canonical car.

The activity area focal to the present study was the Gravity Racer activity. The Gravity Racer included a supply table where groups gathered pre-cut wheels, axles, and cardboard to create the vehicle body. Icon-based instructions (see Figure 1) demonstrated how to make a vehicle with the provided materials. Nearby, the inclusion of a ramp was an intentional design choice meant to spur engineering design cycles within the activity. The ramp accommodated multiple vehicles at a time, featuring lanes of different heights and texture. A simple lever released the vehicles down the ramp.

### 2.2. Data collection and analytical approach

The museum makerspace hosted Cardboard Family Night Events in partnership with community organizations that served primarily families that identified as Black, Indigenous, People of Color (BIPOC). About 90% of adults who attended a Cardboard Family Night Event identified as BIPOC. All adults attending Cardboard Engineering Family Night events were invited to participate in a brief survey about their group’s experience in the makerspace, their own interest in making, and demographic items.

Families were given the option to participate in video-recording their interactions in the makerspace. Groups were given GoPro Hero 4 or GoPro Hero 8 cameras, with tripod mounts. Families were encouraged to turn on and off the camera as they wished, with a goal of capturing at least 20 minutes of video. In addition, follow up interviews were conducted and video-recorded, capturing participants’ reflections of their experience in the makerspace. The combination of survey, video data of time in the makerspace, and follow up interviews with participating families allowed us to develop case studies with a subset of families.

In general, we approached the analysis seeking triangulation across data sources to support trustworthiness of the findings (Shenton, 2004; Carter et al., 2014). Moreover, in the case of video data, video data sessions allowed all authors to contribute to the interpretation of emerging findings (Jordan and Henderson, 1995; Huma and Joyce, 2022). Survey and interview data that supported the key findings from the video data bolstered confidence in the findings that emerged from the cases described below.

#### 2.2.1. Analysis of creativity

The Gravity Racer icon-based instructions depicted a canonical car. It had two axles, four equally sized and balanced wheels, and a canonical car shape. Given that one concern around assembly-style activities is that makers will simply “copy” the instructions, we examined video across groups for products of the Gravity Racer activity. We operationalized creativity as a willingness to diverge from the plans laid out in the Gravity Racer instructions.

#### 2.2.2. Case selection: Novice makers

To examine how Gravity Racer worked for families less comfortable with making, we identified adults that were in the bottom quartile for interest in making on the event survey, meaning they endorsed mostly “No” or “Kind Of” to questions about their enjoyment of broad making activities at home (e.g., fixing things, doing crafts). From there, we identified the ‘Noticing Stations’ case which involved a mother, Deja and her three children Jada (age 9), Lela (age 6), and Zuri (age 2). We selected this case because the caregiver reported not being personally interested in making on the event survey, and in an interview Deja said of being creative, “*My children yes, me no – do not like it, I’d rather read a book, watch a movie, but I have little girls that wants to decorate which actually we just did it the other day [at home].*” We focused on how the group got started in the space and how they approached making from a stance of creativity and engineering thinking.

#### 2.2.3. Case selection: Engineering design process

Similarly, to examine engineering design processes we sought to identify a family that interacted with the Gravity Racer ramp. We identified the ‘No-body Car’ case which involved a set of parents, Kao and Mai and their four children, Eve (age 6), Tou (age 5), Fue (age 2), and Paj (an infant). Analysis focused primarily on Eve’s design and testing of her ‘No-body car’ which was identified during the analysis of creativity described in section 2.2.1. Eve’s engagement, with support from Mai allowed for examination of their entry into the activity, the creativity of the No-body design, and the engineering design processes present within the context of the assembly-style activity.

## 3. Results

### 3.1. Creativity in assembly-style making

**Figure 2:**
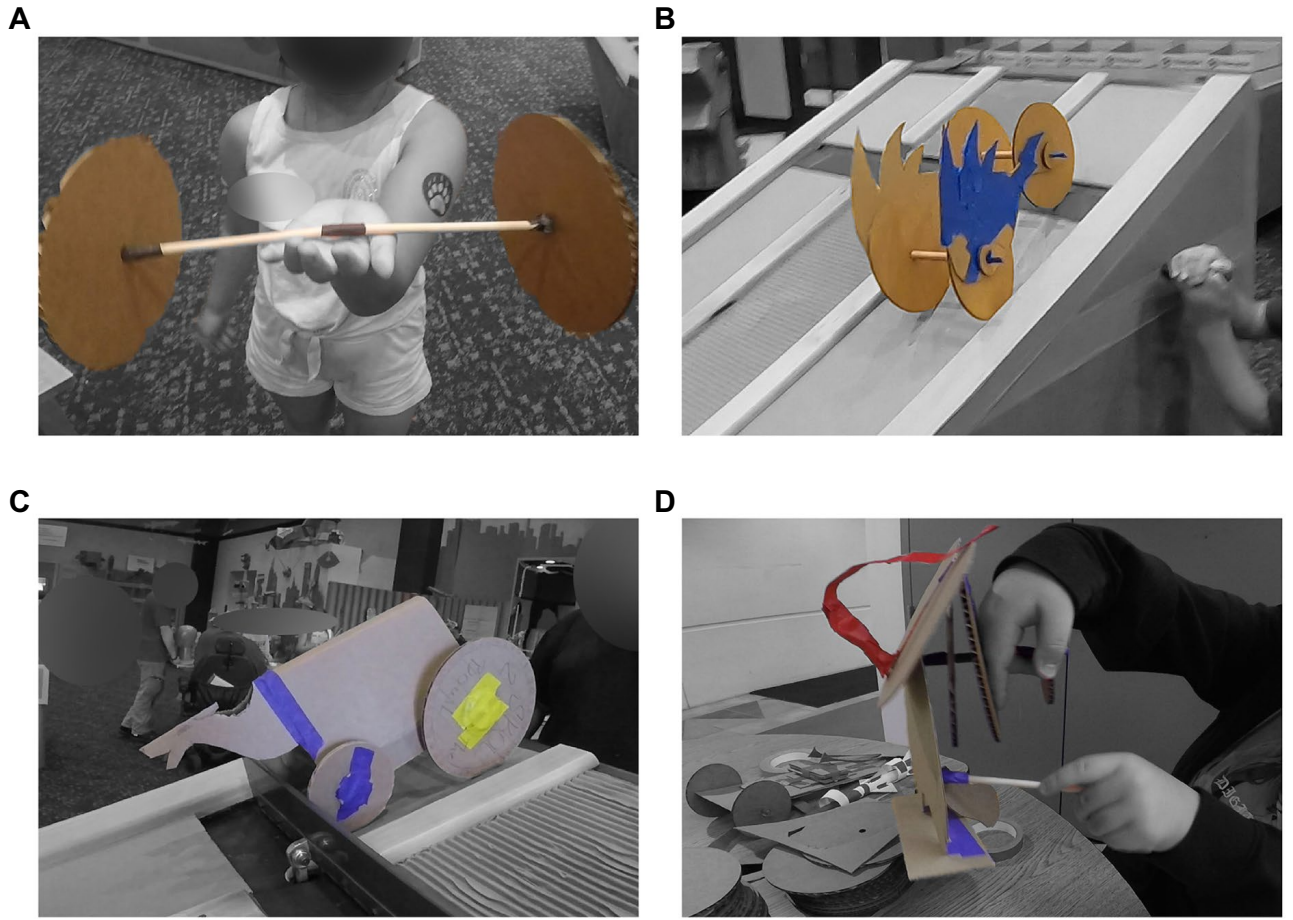
Creative Products of Gravity Racer. These examples highlight just some of the creative variation seen across groups. Panel **(A)**: No-body car (differs in axles), Panel **(B)**: Sonic car (differs in body shape), Panel **(C)**: Viking car (differs in wheel configuration), Panel **(D)**: Puppet (complete remix of materials).

We first sought to explore criticism of assembly-style activities through the lens of creativity. Figure 2 captures examples of products of the Gravity Racer activity, documenting the ways in which they differed from the canonical vehicle included in the instructions. Importantly, across the families that participated in the video research when Gravity Racer was present (*n* = 24), we see variation across their creations in terms of the number of axles, configuration of the wheels, shape of the vehicle body, and in one example a complete re-mixing of the Gravity Racer materials to make a ‘puppet’ character. Thus, providing icon-based instructions suggestive of a canonical car did not limit visitors to just making copies of the suggested design. In fact, though each product of making in Figure 2 highlights a particular feature, the products shown vary across multiple dimensions from the support given in the instructions. In this way, we saw visitors making personally meaningful creations even within the frame of an assembly-style activity.

### 3.2. Noticing stations case

With evidence that creative expression was possible within the Gravity Racer activity, we turned to a potential benefit of assembly-style activities: support for those less comfortable with making. In reflecting on their time in the space as a family, Deja self-identified her role as a supporter of her creative children, saying *“I do not feel like [.] I do not know creativity in that aspect, like building something, just does not flow naturally to me, so that’s why like I’m a good supporter.”*

**Figure 3:**
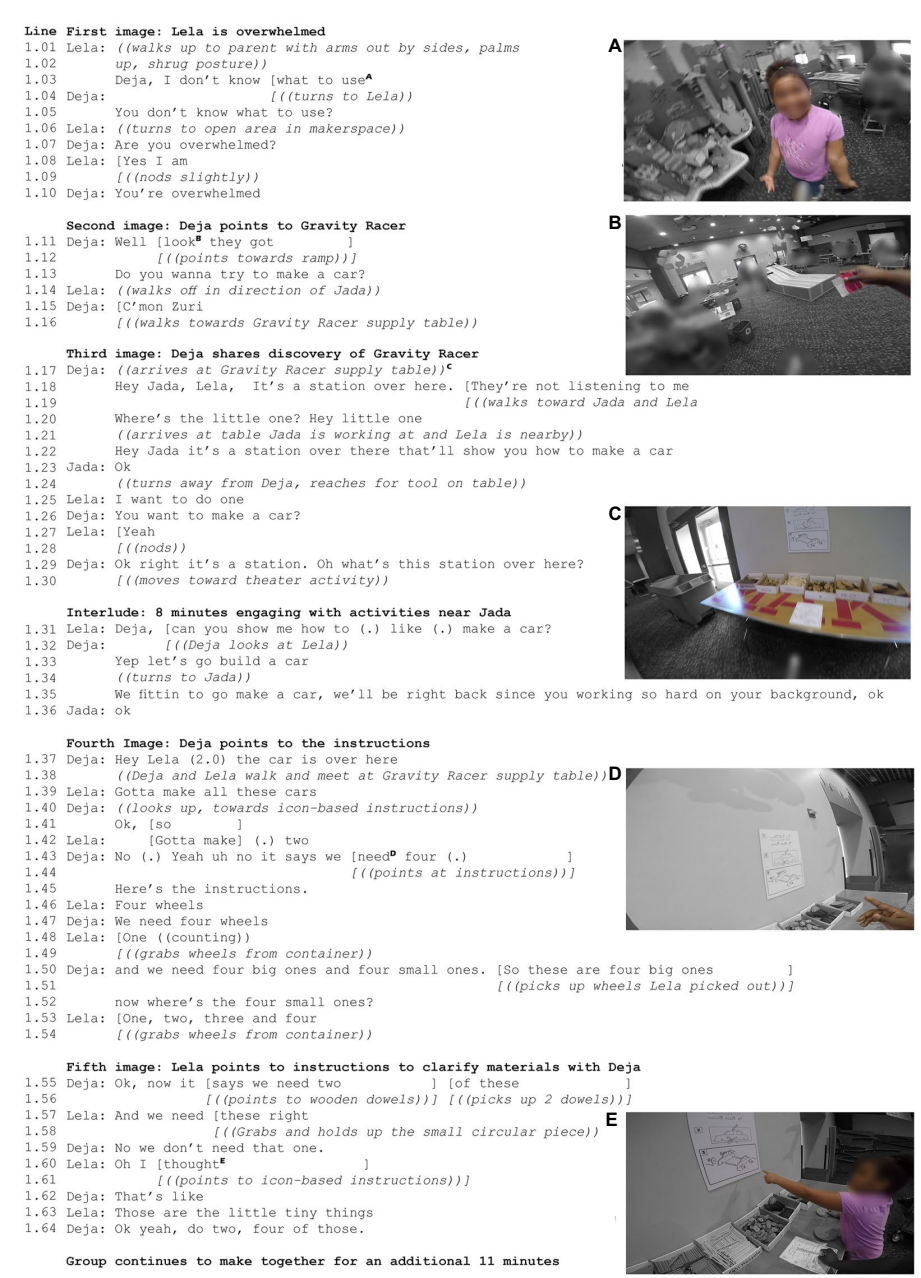
Key Moments in the Noticing Stations Case. Panels **(A–E)** display still images from the video of the group’s entry into the makerspace, alongside a transcript of the group’s verbal interactions that align with these frames.

In Figure 3 we trace the family’s entry into the makerspace focusing particularly on Deja and Lela as the two family members that spent the most time with the Gravity Racer activity.

In line 1.05 (which occurred approximately one minute after Deja began to walk through the space) Lela expressed apprehension about “what to use,” which the adult verbally labeled as “overwhelmed.” In responding to this need, Deja immediately pointed to and then approached Gravity Racer (Figures 3B,C). Multiple elements – the size of the ramp, the supply table, the instructions on the wall – may have supported the adult in noticing this “station” (Figure 3, line 1.18) which offered a path to alleviate feelings of not knowing “what to use” (line 1.05) in the space. Lela expressed interest in making a car, but the group did not immediately act on this interest as they considered other activities.

After several minutes, Lela took up Deja’s offer to make a car (line 1.31). In moving back to the supply table (Figures 3D,E), the icon-based instructions mediated Deja and Lela getting started together, with each pointing towards the assembly-style instructions to begin to collect the materials for making a car. Furthermore, it is in this approximately 12 minutes of making together that Deja begins building her own car. Deja suggested and then enacted a change from the canonical car design to trace her own hand while Lela and Zuri follow her lead. Thus, while Deja reported feeling relatively uncomfortable with being creative, it is in the assembly-style activity where we see Deja take on the role of maker; and problem solve for solutions in making the body of the vehicle.

That is not to say assembly-style making in Gravity Racer completely resolved points of tension for those less comfortable in making. When asked if the activities felt like engineering Deja responded,

“It did, and I was aggravated. I was trying to make a car and for the life of me I do not know how to trace. Yeah, I cannot draw. [audio cuts out] And I was like, I was really thinking, man, people who have to build stuff, I commend them.”

Nonetheless, the ease with which the group noticed the Gravity Racer activity, pointing to it (line 1.12) to alleviate Lela’s feelings about “what to use” and then working together through the accompanying instructions suggests that this assembly-style activity did support this group in having a way to get started in the makerspace.

### 3.3. No-body car case

**Figure 4:**
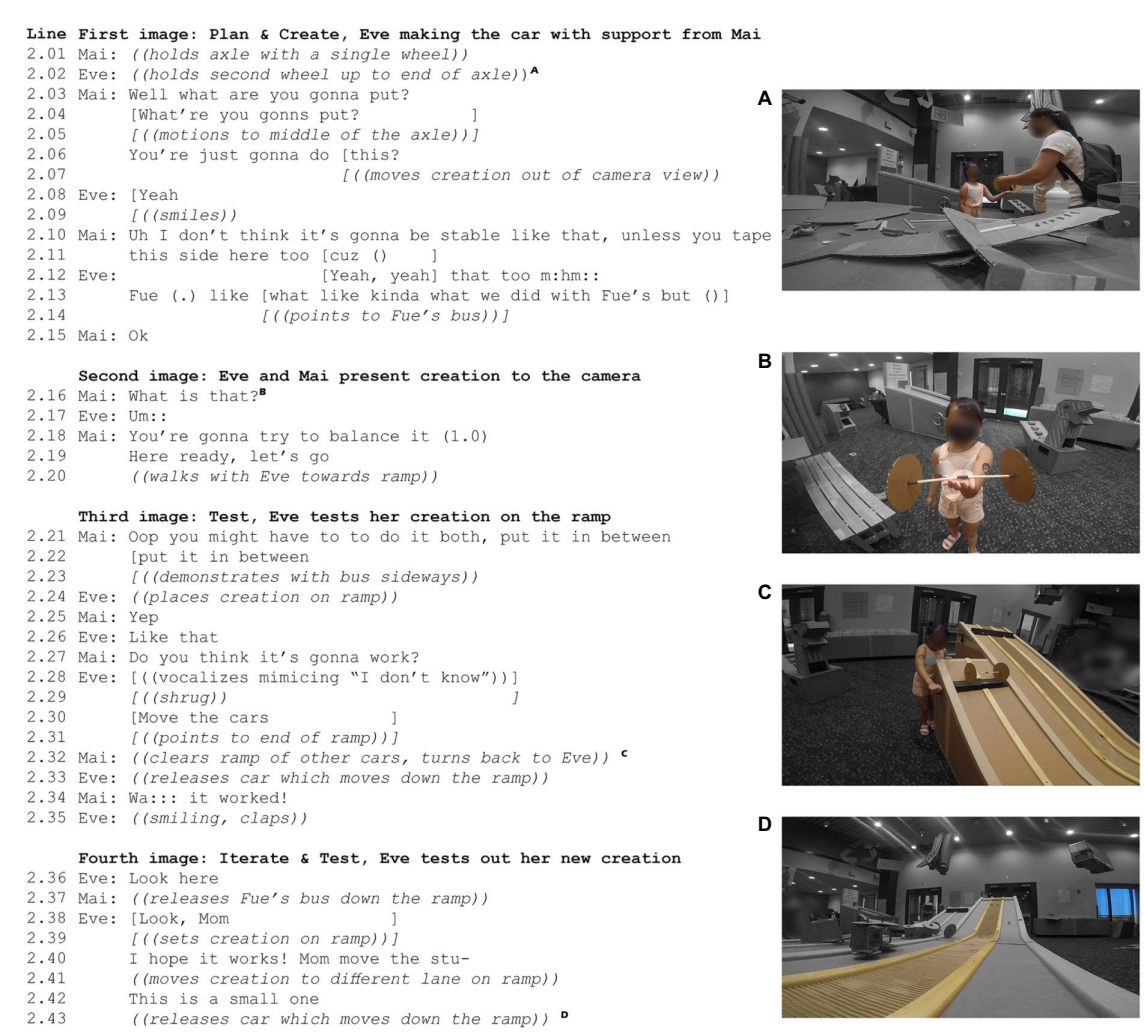
Key Moments in the No-body Car Case. Panels **(A–D)** display still images from Eve’s interactions with Gravity Racer, alongside a transcript of the group’s verbal interactions that align with these frames.

We next turned our focus to engineering design processes. Eve’s car was highlighted in Figure 2 as an example of creative design. Figure 4 traces Eve as she went through an engineering design process – plan, create, test, and iterate. For Eve, the plan and create steps happened intuitively. From the video data available, there was no recorded sequence of her family interacting with the instructions. Instead, the ramp and example vehicles available in the space left behind by other visitors hinted at the possibility of making vehicles. In Mai’s interview she recalled,

“.. [Eve] noticed that my boys were playing with the ramp. She wanted to come up with something that would roll down the ramp. And then it got to where she wanted to see whose is the fastest, or what can she build that can go down the ramp the fastest, kinda like a race. So, that's why everyone just turned their attention to the ramp of, hey, I wanna build something. I wanna see how fast it can go. I wanna build the fastest.”

Eve’s goal displayed an engineering mindset in building for efficiency. We investigated this case further by considering parallels to engineering design processes.

Mai supported Eve in her planning and creation, though she voiced some skepticism about the plan to include no vehicle body in the design (Figure 4, line 2.06). Following the completion of the creation stage, Eve tested her design with support (Figure 4, lines 2.21–2.35). Eve later tested the no-body car another time, with Fue grabbing the car at the end of the ramp. This action prompted Eve to collect supplies to make a smaller iteration of the same design and test that iteration (Figure 4, lines 2.36–2.43). Thus, the case evidenced many instances of engineering thinking and demonstrated that the entirety of the engineering design process (plan, create, test, iterate) were possible within the Gravity Racer activity.

## 4. Discussion

The present study sought to explore assembly-style making along dimensions of 1) creativity, 2) support for novices, and 3) engineering thinking. We developed case studies around two families interacting with a cardboard-focused makerspace in a museum setting. Given concerns that assembly-style activities, like Gravity Racer, that include instructions would eliminate creativity and limit engineering thinking, rich video counterexamples provided compelling evidence that assembly-style activities may play an important role when considering the mix of activities available to families in makerspaces.

Revisiting the learning dimensions possible in makerspaces, we found that groups captured many instances of creative expression while they were engaged in an assembly-style activity. Our findings serve to blur the lines between categories within Bevan’s (2017) taxonomy of maker activities. The instructions for Gravity Racer and pre-cut wheels and axles lend the activity to assembly-style forms of making, but some aspects of the activity such as the fashioning of the vehicle body lean more towards creative construction. In the ‘Noticing Stations Case’ Deja suggested and then enacted a creative solution of tracing her hand to make the body of the vehicle. Likewise, Eve took a creative approach in the ‘No-body Car Case’, designing a vehicle with only one axle and no car body. Even with instructions present in the space (and Deja and her family attempting to follow the instructions closely) groups engaged creatively in their making.

We were interested in how an assembly-style activity, like Gravity Racer, might support novices in the space. For this study, we defined novices as individuals who self-reported primarily “No” or “Kind of” when surveyed on their enjoyment of making (building things, fixing things, etc.). By focusing on a group in the ‘Noticing Stations Case’, we noted how Deja responded immediately to Lela feeling “overwhelmed” by pointing to the Gravity Racer activity. Later, when they take up making together the instructions mediated interactions between caregiver and child, with each pointing to and gathering materials together. That is not to say that having instructions present in the space means that everyone seeks them out and follows them. Eve relied mainly on example pieces, which have also been discouraged in the maker literature (e.g., MakerEd, 2015). This group never interacted with the instructions on camera (groups were free to turn the camera off and did so over the course of their time in the space). This suggests that while the Gravity Racer clearly lends support consistent with an assembly-style mode of making, makers may take on tasks more consistent with creative construction or even tinkering over the course of making in a free choice space. Instructions provide one way of getting started but not the only way to approach the activity.

Groups engaged with the Gravity Racer also engaged in engineering thinking. Deja and Lela used the instructions to plan, and then had to iterate and problem solve around ways to make the body of the car. Eve tested multiple creations on the ramp alongside her family members. This particular assembly-style activity, Gravity Racer seemed ripe for fostering skills related to engineering thinking.

One advantage of making with a widely available material like cardboard was that groups could continue making at home. In fact, we heard from several groups involved with the larger study that their children had continued making with cardboard at home– including from Deja and Mai. We are less certain how caregivers who are less confident about making (as in the ‘Noticing Stations’ case) might engage in making at home, with or without the use of icons and instructions for support. This suggests a productive line of inquiry for future research into assembly-style activities.

In recent years, makerspaces have been seen as opportunities to advance equity in informal learning settings (Calabrese Barton et al., 2017). This analysis was part of a larger project centering BIPOC family experiences in making. While the present study utilizes that data, we were not aiming to make a claim about BIPOC families in particular. Several studies on equity in makerspaces focus on youth working with educators (e.g., Calabrese Barton et al., 2017; Sengupta-Irving and Vossoughi, 2019). More work could consider family interactions in makerspaces, building off insights around creativity or engineering thinking in the present study, or the work of other researchers considering family learning (e.g., Tzou et al., 2019).

Our analysis is limited in that the case selection focused on just two families that in some ways represented a best-case-scenario perspective on features of interest, namely creativity, getting started in the makerspace, and engaging in engineering design processes. This approach was warranted given that most advice in maker education prioritizes tinkering over assembly-style activities. Further replication with other assembly-style activities would bolster confidence in the strengths and weaknesses of assembly-style making. A second potential limitation of the present study was that the assembly-style activity directly included a clear means to test one’s creation in the form of a ramp. We hypothesize that including this ‘test bed’, designed to be both fun and encourage iterations, is an important part of noticing the potential of the activity and offering multiple entry points into the engineering design process. Future studies might consider if the evidence supporting assembly-style making is as strong without such a designed element present.

We conclude that makers may benefit from a mix of activities being available in the makerspace. While the literature elsewhere has shared the benefits of tinkering, the present study demonstrated that providing assembly-style activities may address visitors’ comfort in making and alleviate potential hesitancy in how to start. Makers can be creative and practice or engage in engineering thinking within the frame of an assembly-style activity. We encourage practitioners to tinker with assembly-style activities in their own spaces, see how makers use those activities to get started, and to go farther than the directions. We also encourage other researchers to look at these activities to understand how they operate in a space – for instance, how do families with multiple levels of experience, or multiple interests, use activities like these? Are some assembly-style activities structured in ways that support less or more creativity, or work worse or better as ways to get started in the space? Our exploration of assembly-style activities suggests that they are appropriate to include, but more can be understood about the many roles assembly-style making can play in a multi-generational makerspace.

## Data availability statement

The original contributions presented in the study are included in the article/supplementary material, further inquiries can be directed to the corresponding author.

## Ethics statement

The studies involving human participants were reviewed and approved by Heartland Institutional Review Board. Written informed consent to participate in this study was provided by the participants’ legal guardian/next of kin. Written informed consent was obtained from the individual(s), and minor(s)’ legal guardian/next of kin, for the publication of any potentially identifiable images or data included in this article.

## Author contributions

MB, MG, and SL contributed to the conceptualization and design of the study. BS led the concept development and design of Cardboard City. SL led the analysis and prepared the first draft of the manuscript. MG and SL prepared the figures. All authors contributed to video data analysis and manuscript revision, all read and approved the submitted version.

## Funding

This material is based upon collaborative work supported by the National Science Foundation under Grant no. 1906884, Building More Inclusive Makerspaces to Support Informal Engineering Learning Experiences. Any opinions, findings, and conclusions or recommendations expressed in this material are those of the authors and do not necessarily reflect the views of the National Science Foundation.

## Conflict of interest

The authors declare that the research was conducted in the absence of any commercial or financial relationships that could be construed as a potential conflict of interest.

## Publisher’s note

All claims expressed in this article are solely those of the authors and do not necessarily represent those of their affiliated organizations, or those of the publisher, the editors and the reviewers. Any product that may be evaluated in this article, or claim that may be made by its manufacturer, is not guaranteed or endorsed by the publisher.
